# Hydrogels and Bioprinting in Bone Tissue Engineering: Creating Artificial Stem‐Cell Niches for In Vitro Models

**DOI:** 10.1002/adma.202301670

**Published:** 2023-11-02

**Authors:** Francesca K. Lewns, Olga Tsigkou, Liam R. Cox, Ricky D. Wildman, Liam M. Grover, Gowsihan Poologasundarampillai

**Affiliations:** ^1^ School of Dentistry University of Birmingham Birmingham B5 7EG UK; ^2^ Department of Materials University of Manchester Manchester M1 5GF UK; ^3^ School of Chemistry University of Birmingham Birmingham B15 2TT UK; ^4^ Faculty of Engineering University of Nottingham Nottingham NG7 2RD UK; ^5^ Healthcare Technologies Institute School of Chemical Engineering University of Birmingham Birmingham B15 2TT UK

**Keywords:** 3D bioprinting, bone tissue modeling, extracellular matrices, hydrogels, in vitro models

## Abstract

Advances in bioprinting have enabled the fabrication of complex tissue constructs with high speed and resolution. However, there remains significant structural and biological complexity within tissues that bioprinting is unable to recapitulate. Bone, for example, has a hierarchical organization ranging from the molecular to whole organ level. Current bioprinting techniques and the materials employed have imposed limits on the scale, speed, and resolution that can be achieved, rendering the technique unable to reproduce the structural hierarchies and cell–matrix interactions that are observed in bone. The shift toward biomimetic approaches in bone tissue engineering, where hydrogels provide biophysical and biochemical cues to encapsulated cells, is a promising approach to enhancing the biological function and development of tissues for in vitro modeling. A major focus in bioprinting of bone tissue for in vitro modeling is creating dynamic microenvironmental niches to support, stimulate, and direct the cellular processes for bone formation and remodeling. Hydrogels are ideal materials for imitating the extracellular matrix since they can be engineered to present various cues whilst allowing bioprinting. Here, recent advances in hydrogels and 3D bioprinting toward creating a microenvironmental niche that is conducive to tissue engineering of in vitro models of bone are reviewed.

## Graphical abstract

This review focuses on hydrogels and 3D bioprinting in bone tissue engineering for development of in vitro models of bone. It highlights challenges in recapitulating the biological complexity seen in bone and how synergistic application of dynamic hydrogels and innovative bioprinting pipelines can address these challenges to achieve bone models.

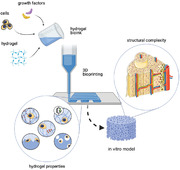

## 1 Introduction

Bone is a highly complex, active, and hierarchically structured tissue formed of different types of bone cells and calcified collagen matrix, structured from the nano‐ to the macroscale.^[^ ^1^, ^2^ ^]^ Its dynamic and vascularized nature means it undergoes constant remodeling.^[^ ^3^ ^]^ Bone can be categorized into two different types of tissue: cortical and trabecular bone, both of which have a rich abundance of vascular supply.^[^ ^4^, ^5^ ^]^ Vascularization plays a key role in bone formation and remodeling processes,^[^ ^6^ ^]^ contributing to the immigration of mesenchymal stem cells (MSCs), the supply of oxygen and nutrients to metabolically active tissues, and the removal of waste by‐products.^[^ ^7^ ^]^ Specific vascular microenvironmental niches also support tissue‐resident unique adult stem cells,^[^ ^8^ ^]^ key to bone formation and remodeling. Without vascularization and specific vasculature, the formation of new functional bone tissue is not possible.

Historically, bone tissue engineering (BTE) has concentrated on regeneration, focusing on creating implants using scaffolds, cells, mechanical stimuli, and soluble factors for patients with significant bone defects. Due to bone's natural ability to regenerate and remodel, tissue‐engineered bone‐like structures can stimulate regeneration even if they do not mimic the intricate ECM structure of real human bone. There is currently a growing interest in the application of BTE for in vitro 3D human models of healthy and pathological bone for drug development and systemic interactions. However, current models are unable to accurately model bone hierarchy and perfusable vasculature. Therefore, the development of more sophisticated models needs to be explored.

Creating an in vitro mimic in which hierarchical structures and properties are replicated wherein the various cell cohorts are assembled and orchestrated to form functional bone remains a major challenge for BTE. The in vitro fabrication of bone tissue requires the use of a material that can support sufficient diffusion of oxygen, nutrients, and cell metabolites to maintain cell viability throughout. Hydrogels are a class of materials that are formed principally of water and an entangled network of polymeric molecules, which enables cell encapsulation and ensures high cell viability. Moreover, it is possible to finely tune hydrogel properties, such as viscoelasticity, to tightly regulate stem cell behavior.^[^ ^9^, ^10^, ^11^, ^12^ ^]^ When hydrogels and their stem cell directive cues are implemented together with 3D biofabrication, there is the potential to take both biological and structural complexity to another level of hierarchy to create a complex architecture that is analogous to that found in bone.

This review provides an overview of the important roles that both hydrogels and 3D bioprinting play in achieving biological and structural complexity in bone tissue mimics and their responsibilities in directing stem cells to recapitulate the stem cell niche.

## 2 The Barriers to Bone Tissue Engineering

**Figure 1:**
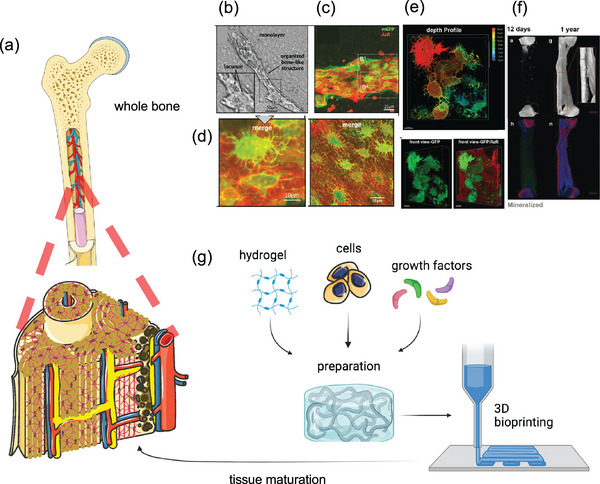
a) Bone structure, from whole bone to cortical and trabecular bone with vascularisation which cannot be recapitulated by current models. Current in vitro models of highly organized bone‐like structures – b) brightfield image and enlarged inset showing a bone‐like structure with c) confocal image of mineralized bone structure at day 28, d) highly dendritic osteocyte‐like networks with osteocytes embedded within mineralized lacunae, e) confocal microscopy shows the 3D bone‐like structures and layers of interconnected osteocytes. Reproduced (Adapted) under the terms of the CC‐BY license.^[^ ^22^ ^]^ Copyright 2020, the Authors. Published by American Society for Bone and Mineral Research. f) Long‐term in vitro model of osteocyte with images demonstrating the development of bone matrix and mineralization over time (12 days to 1 year). Reproduced (Adapted) under the terms of the CC‐BY license.^[^ ^21^ ^]^ Copyright 2018, the Authors. Published by Wiley‐VCH GmbH. g) Schematic illustration of approaches employing hydrogels in BTE, combining hydrogels, e.g., natural or synthetic, cells, e.g., mesenchymal stem cells, and growth factors, e.g., bone morphogenetic protein 2, with 3D bioprinting to lead to tissue maturation. Created on the BioRender website (https://biorender.com) by the authors.

BTE requires several components, including stem cells, appropriate stimuli, and scaffolding, along with a manufacturing process to assemble these components, and a tissue‐maturation process that allows self‐organization into a complex architecture whilst also enabling vascularization (**Figure** **1**). Thus, dynamic presentation of biophysical and biochemical cues to direct bone formation and remodeling via scaffolds and signals must be homogenized with both the manufacturing process and the maturation phase. At present, it is possible to engineer in vitro several structures of bone hierarchy individually.^[^ ^1^ ^]^ Iordachescu and colleagues created a micro‐organ model that functions as a complete unit that can detect and recapitulate in fine detail the early stages of cellular processes, including bone resorption and the subsequent osteo‐proliferation and mineral deposition (Figure 1).^[^ ^13^ ^]^ Also, it has also been possible to create an organoid in vitro model that mimics osteogenesis both in physiological and pathological situations, as demonstrated by the Iordachescu and Hofmann groups.^[^ ^13^, ^14^ ^]^ Reader is directed to reviews involving co‐culture of osteoblasts and osteoclasts to generate bone.^[^ ^15^, ^16^ ^]^

Despite recent advances,^[^ ^17^, ^18^, ^19^, ^20^, ^21^, ^22^ ^]^ there remain obstacles to functional BTE. BTE has not delivered tissue mimics containing mature lamella bone together with the appropriate vascular tree structure and bone marrow (Figure 1a). This is primarily due to three principal barriers:

1) Difficulty in recapitulating bone architecture and organization, and its dynamic remodeling processes,
2) Difficulty incorporating functional and mature vascularization,
3) Difficulty in delivering appropriate organ‐level stimulation (mechanical loading and fluid flow).

To bridge the gap between engineered and native tissues it is important to overcome the limited spatial resolution barrier associated with 3D biofabrication strategies and to innovate to produce complex hierarchical architectures with hosts of cells and material formulations (Figure 1g).^[^ ^23^ ^]^ The processes (signals – chemical, physical, and mechanical) involved in constructing specialized microenvironments through to cellular phenotypic effects using material formulations and biophysical cues should also be considered synchronously when investigating biological and structural functionality.

## 3 Biomaterial Challenges: Material Properties

**Figure 2:**
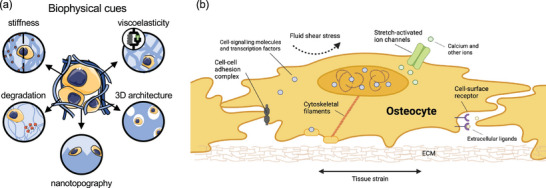
a) Schematic of biophysical cues of microenvironment for cell behavior, including stiffness and viscoelasticity, which are involved in mechanotransduction, degradation, and 3D architecture, including nanotopography. b) Cellular processes of mechanosensing and response with several biological components, not mutually exclusive, depicted in a bone cell such as an osteocyte. Created on the BioRender website (https://biorender.com) by the authors.

The ECM supports and influences important cellular processes including morphology, migration, and fate. Cells sense and modify their matrix in response to biochemical and biophysical stimuli (**Figure** **2**a), as well as oxygen levels and nutrient concentration, all factors that contribute to the microenvironmental niche.^[^ ^24^, ^25^, ^26^ ^]^ Mechanosensitive feedback is important in bone tissue, as osteoprogenitors, but also bone cells, such as osteocytes, osteoblasts, and osteoclasts, alter their phenotype and function as a result of external physical and fluid forces from the surrounding environment to regulate tissue formation (Figure 2b).

Hydrogels show great promise for BTE from both a biological and an engineering standpoint.^[^ ^27^, ^28^ ^]^ Their ability to mimic the fibrous structure of natural ECM, their pliability, high processability, and porous structure, enabling oxygen and nutrient exchange whilst supporting cell attachment, proliferation, and differentiation, make them a “go to” class of material for BTE.^[^ ^28^ ^]^

The body of literature reviewing hydrogels is extensive and the reader is directed to excellent reviews on compositions^[^ ^29^, ^30^, ^31^ ^]^ and properties^[^ ^32^, ^33^, ^34^ ^]^ for BTE. Therefore, the following section explores hydrogel biophysical cues such as mechanics, architecture, and degradation in regulating in vitro cell behavior, namely: 1) cell attachment and organization; 2) cell proliferation and differentiation; and 3) tissue formation and remodeling. It is important to note that the hydrogel biophysical properties are highly interdependent and thus varying one will influence the other; thus, it is challenging to isolate the effect of one variable over the other on cell behavior. In this section, we attempt to identify those biophysical properties that have the greatest effect on each of the key cell processes in bone formation and remodeling.

### 3.1 Cell Attachment and Organization

Bone formation in vitro involves the delivery of stem and/or specialized cells, with or without appropriate signaling cues, often employing a supporting scaffold.^[^ ^35^ ^]^ Delivery does not necessitate cell attachment; however, bone cells, and specifically osteoblasts, are adherence‐dependent cells, rendering regulation of cell attachment an exigency for subsequent cellular function. Thus, promotion of cell attachment to a hydrogel matrix via biochemical and biophysical cues is a key aspect to optimize. The biochemical composition of a hydrogel is an important factor that controls cell attachment, and in some cases, biochemical cues can dominate over biophysical cues at this stage of BTE.^[^ ^36^, ^37^ ^]^ Biochemical cues, including ligand chemistry, cryptic peptides, bound growth factors (GFs), and extracellular vesicles are mostly used to enhance cell attachment. The literature on biochemical cues is extensive and the reader is directed to other reviews for more information.^[^ ^38^, ^39^, ^40^ ^]^ Whilst hydrogels can present cell‐attachment motifs, such as the arginine‐glycine‐aspartate (RGD) peptide, and deliver GFs, 3D bioprinting has the potential to facilitate the production of BTE constructs with spatiotemporally defined patterns of GFs to promote the organization and self‐assembly^[^ ^41^ ^]^ of specific cells to stimulate bone tissue formation, vascularization, and remodeling.

#### 3.1.1 Role of Hydrogel Architecture in Cell Attachment and Organization

Nanostructures, such as nanoparticles, have been used to control cell attachment and organization^[^ ^42^ ^]^ through manipulation of cell exposure to motifs.^[^ ^43^ ^]^ RGD‐functionalized silica nanoparticles were conjugated onto a thiolated methacrylated hyaluronic hydrogel. The 1.3‐fold increase in the percentage of initial cell attachment when silica nanoparticles were used on a methacrylated hyaluronic hydrogel when compared to no nanoparticles,^[^ ^43^ ^]^ highlights how unique surface topographic inputs can be used as directive cues for cells. Bioorthogonal strategies have been employed to crosslink and encapsulate cells within controlled nanostructured hydrogel networks.^[^ ^44^ ^]^ Nanostructures have also been used within DNA hydrogels. A rapidly formed supramolecular polypeptide–DNA hydrogel was prepared and used for in situ multilayer 3D bioprinting.^[^ ^45^ ^]^ Designed structures were printed using two complementary bio‐inks that were alternately deposited. This bioprinter‐based method created various 3D tissue‐like patterns and structures with the necessary scales and dimensions, through varying droplet sizes and the number of layers printed.^[^ ^45^ ^]^ Additionally, DNA hydrogels have been used to create large, centimeter‐scale constructs with excellent hemo‐ and cytocompatibility.^[^ ^46^ ^]^ For further information regarding structural DNA nanotechnology, the reader is directed to a comprehensive review.^[^ ^47^ ^]^

As described before, cell attachment and organization are fundamental requirements for further key cellular processes involved in bone formation in vitro. The architecture of hydrogels, including nanostructures and molecular assembly, is a biophysical cue that dominates at the early stages of the bone formation process and can be exploited to promote and optimize cell attachment and organization for the next stage, namely, cell growth.

### 3.2 Cell Growth: Migration, Proliferation, and Differentiation

Variations in hydrogel mechanics, such as stiffness and viscoelasticity, and altering the 3D architecture, such as pore size and nanotopographical features, affect and instigate changes in cell behavior, activity, and phenotype. Stiffness and viscoelasticity are key mechanical properties that are converted into biochemical signals in a process called mechanotransduction. Mechanosensitive feedback is particularly important in bone tissue, as bone cells, such as osteocytes, osteoblasts, and osteoprogenitors, alter their phenotype and function in response to external physical forces from the surrounding environment, to regulate tissue formation and remodeling.^[^ ^48^ ^]^ Several approaches have been explored to control cell migration, proliferation, and differentiation in hydrogels. Work has been focused on mechanotransduction; thus, stiffness and viscoelasticity will be discussed first. An overall summary of biophysical cues covered in the following sections is presented in Figure 2a.

#### 3.2.1 Role of Hydrogel Stiffness in Cell Migration, Proliferation, and Differentiation

In vivo cell migration is attributed to chemotaxis; however, in vitro, cells can also follow a gradient in mechanics, also known as durotaxis.^[^ ^49^ ^]^ Tugging traction dynamics within cell‐ECM focal adhesions (FAs) are key to durotaxis. This sensing mechanism is capable of operating over a broad range of matrix stiffness and tension, such that directional migration of cells can be tightly controlled along highly localized or dynamically changing ECM‐rigidity gradients.^[^ ^50^ ^]^

**Figure 3:**
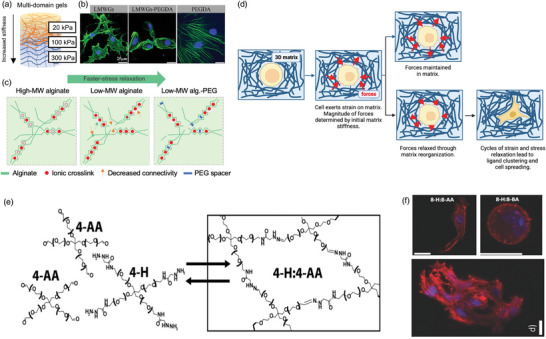
a,b) Effect of hydrogel stiffness and c–f) viscoelasticity on cellular response. a) A biomimetic gradient for osteochondral tissue regeneration, b) MSCs stained for actin cytoskeleton on three different gels: LMWGs, LMWG‐PEGDA, and PEGDA gels for 14 days. Reproduced (adapted) with permission.^[^ ^51^ ^]^ Copyright 2021, Elsevier. c) Schematic showing molecular weight (MW) of alginate crosslinked by calcium with entanglement and connectivity of the network, with both molecular weight and PEG spacers increasing the rate of stress relaxation. d) The effect of a 3D matrix on a cell – resulting in forces and stresses acting on the cell. When forces are maintained, there is no remodeling, whereas, if there are cycles of stress and strain relaxation it results in cell spreading.^[^ ^62^ ^]^ e) Chemical structure of an aliphatic hydrazone‐linked hydrogel, showing reversible gelation. f) Dynamic nature of the aliphatic hydrazone‐linked hydrogel results in filopodia and lamellipodia extension, facilitating cytoskeletal outgrowth. Reproduced with permission.^[^ ^70^ ^]^ Copyright 2014, Wiley‐VCH GmbH.

There is a close relationship between hydrogel stiffness and cell migration and differentiation.^[^ ^51^ ^]^ He et al.,^[^ ^51^ ^]^ created a gel to engineer osteochondral tissue with an increasing stiffness gradient from the cartilage (20 kPa) to the bone layer (300 kPa) (**Figure** **3**a). The different domains had the capacity to induce differentiation of MSCs toward the chondrocytic and osteoblastic lineages (Figure 3b). Although stiffness‐dependent mechanoregulation impacts many cellular processes, its impact on stem‐cell spreading, differentiation, and migration has been clearly demonstrated.^[^ ^52^, ^53^, ^54^, ^55^ ^]^ Additionally, cell proliferation has been shown to have a biphasic dependence on matrix elasticity, peaking in intermediate stiffness gels (60 kPa), which also supported osteogenic lineage commitment.^[^ ^56^ ^]^ Matrix stiffness plays a key role in regulating MSC differentiation into specific mature cell types. Dynamic modulation of stiffness can result in the activation of different transcription factors that upregulate genetic pathways (YAP/TAZ). These pathways are responsible for the initiation and progression of particular cell‐lineage differentiation.^[^ ^57^ ^]^ The genes responsible and the pathways involved are beyond the scope of this review, so the reader is directed to another comprehensive review for details.^[^ ^57^ ^]^

#### 3.2.2 Effect of Hydrogel Viscoelasticity on Cell Migration, Proliferation, and Differentiation

Natural tissues and cells themselves are viscoelastic.^[^ ^58^, ^59^ ^]^ Cells probe their environments at several frequencies.^[^ ^60^ ^]^ Influence of hydrogel viscoelasticity in directing cell phenotype and migration has been covered by Chaudhuri, Mooney, and co‐workers.^[^ ^59^, ^61^, ^62^, ^63^, ^64^ ^]^ Within viscoelasticity and cell migration, literature often contradicts. Early studies^[^ ^65^, ^66^ ^]^ suggested that decreased viscosity and increased stiffness promoted cell spreading through cell traction forces. However, cell spreading has also been reported to be upregulated in 3D viscoelastic matrices. Similarly, viscoelasticity was found to promote and regulate the proliferation of multiple cell types in hydrogels.^[^ ^62^, ^67^ ^]^ Disagreement may arise from difficulty in varying and controlling hydrogel mechanics accurately and independently over other biophysical and chemical cues.

Fast stress relaxation has been shown to allow for better cell spreading in calcium‐crosslinked alginate hydrogels when compared to alginate gels with slow stress relaxation times with similar elasticity (Figure 3c). Mooney and co‐workers showed that fast stress‐relaxing matrices undergo mechanical remodeling to result in adhesion‐ligand clustering, cell spreading, proliferation, and matrix formation (Figure 3d)^[^ ^62^, ^63^ ^]^ Initial moduli of ≈9 kPa or 17 kPa induced MSCs to differentiate into fat and bone, respectively.^[^ ^62^ ^]^ However, the stiffer matrices with faster relaxation kinetics, which are usually characteristic of moving toward a progressively softer modulus, showed an increase in osteogenic differentiation as a function of matrix viscoelasticity. It was hypothesized that the cells sense matrix relaxation in real‐time, rather than integrating the mechanical signal over time.

Bio‐orthogonal covalent crosslinking has been explored together with ionic crosslinking to produce alginate networks, providing a way to control viscoelasticity and stress relaxation times.^[^ ^68^, ^69^ ^]^ Further to this, fibrillar collagen matrix was integrated into the system. The various levels of crosslinking resulted in a range of storage moduli and elastic moduli (1.5–7.5 kPa). Increased elasticity of the matrix, as a result of covalent crosslinks, altered the cells’ response to mechanical cues with respect to regulation of gene expression of immunomodulatory markers by human MSCs (hMSCs).^[^ ^68^ ^]^

**Figure 4:**
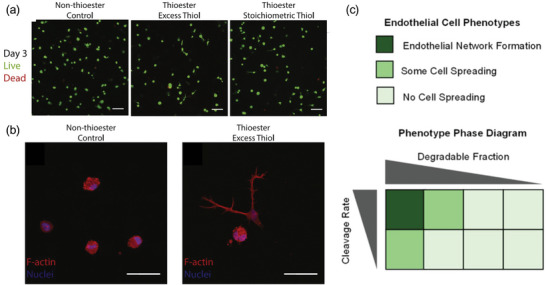
Role of a,b) hydrogel viscoelasticity and c) degradation on remodeling Viscoelasticity, a) Day 3 LIVE/DEAD showing increased spreading in thiol‐containing hydrogels, and b) cell shape on day 3, remaining rounded in the control and spread in excess thiol hydrogels. Reproduced (adapted) with permission.^[^ ^71^ ^]^ Copyright 2023, Elsevier. c) Degradation, with a focus on degradation fraction and cleavage rate on endothelial network formation. Reproduced (adapted) with permission.^[^ ^92^ ^]^ Copyright 2023, American Chemical Society.

Anseth and co‐workers have employed hydrazone crosslinks to produce hydrogels that remain dynamic under physiological conditions.^[^ ^70^ ^]^ The dynamic stress‐relaxing crosslinks allow for rearrangement of the gel over a timescale that is suitable for filopodia and lamellipodia extension (Figure 3e,f).^[^ ^70^ ^]^ Furthermore, Anseth and co‐workers developed a covalent adaptable thioester‐crosslinked hydrogel with viscoelastic properties ranging over several orders of magnitude, by modifying pH, gel stoichiometry, and crosslinker structure. In these adaptable thioester hydrogels, hMSCs were able to spread within 1 day of culture and developed stellate morphologies in contrast to the rounded shape of cells observed in the static, control hydrogels lacking the thioester functionality. This study highlights the potential of adaptable crosslinking strategies to mimic ECM viscosity, and the use of these crosslinking strategies within hydrogels to remodel their surroundings (**Figure** **4**a,b).^[^ ^71^ ^]^

Knowledge of how cells sense, interpret, and react to changes in mechanical signals on timescales ranging from seconds to hours is key in improving the design of dynamic hydrogels for cell growth.^[^ ^72^ ^]^ Additionally, mechanical signals will vary significantly due to the formation of precellular matrix and hydrogel degradation over a timescale of hours to weeks, respectively. It is therefore important to design hydrogels and implement strategies that exploit the cell‐mediated dynamical changes across a large time scale.

#### 3.2.3 Role of Hydrogel Degradation in Cell Migration, Proliferation, and Differentiation

Matrix degradation is a key factor that influences cell motility and migration,^[^ ^73^ ^]^ discussed in detail in Section 4.3.1. It was found that in poly(ethylene glycol)‐ (PEG) based hydrogels, cell migration is independent of proteolytic degradation within a low‐stiffness matrix, whereas, at higher levels of matrix stiffness, proteolytic migration dominated.^[^ ^74^ ^]^ Further, hMSC migration decreased with increasing stiffness in a matrix metalloproteinase‐ (MMP) degradable, peptide‐functionalized PEG hydrogel.^[^ ^75^ ^]^ The cell‐mediated degradation of the pericellular matrix increased with increasing post‐encapsulation time. This observation suggests that over time, hMSCs secrete MMPs to degrade and remodel the matrix to enable migration, with relatively higher rates in softer matrices than stiffer ones. In addition to higher stiffness, a high density of matrix (i.e., polymer concentration) is also known to reduce the speed of cell migration.^[^ ^74^, ^76^ ^]^ Both higher stiffness and density of matrix had the effect of limiting the space available or the geometry of pores for cell migration. Cell migration is therefore facilitated by proteolytic degradation;^[^ ^77^ ^]^ however, degradation should be tuned with remodeling. Thus, hydrogel network geometry and architecture are other factors that influence cell movement in 3D hydrogels and must therefore be optimized together with mechanics and degradation to facilitate cell growth, organization, and assembly.^[^ ^78^ ^]^

#### 3.2.4 Role of Hydrogel 3D Architecture in Cell Migration, Proliferation, and Differentiation

3D Hydrogel mesh size or confinement heavily influences cell motility and migration. Literature reporting hydrogel mesh size on cell growth is sparse due to the difficulty in measuring and controlling hydrogel mesh size in 3D. The main take‐home message from the literature is that the nucleus acts as the main steric hindrance when cells migrate in confined 3D spaces.^[^ ^79^ ^]^ In addition, cell‐migration speeds are highest in intermediate channel widths (12 µm). Multiple cell types were confined in spaces smaller than the individual cells, but bigger than the nucleus; cells displayed the fastest migration speeds through either porous scaffolds or 3D channels.^[^ ^79^, ^80^ ^]^ Additionally, 3D cell migration in the absence of proteolytic degradation is significantly limited when pore diameter reaches below 4 µm, which is comparable to the size of most cell nuclei.^[^ ^79^, ^81^ ^]^ At these levels of confinements, pore size dominates over substrate matrix stiffness in controlling migration.

Nanofibrillar structures, formed from gellan gum hydrogels and arranged by thermally driven self‐assembly, facilitated both cell adherence and proliferation.^[^ ^82^ ^]^ Increased adherence and proliferation were hypothesized to originate from the change in density and pore size decrease from thermal annealing, which resulted in microstructural rearrangements.^[^ ^82^ ^]^ Hydrogels held at 65 °C for 16 h exhibited the best results for cell adhesion and proliferation. The nanotopography further influenced hydrogel stiffness, with an increase in local stiffness observed in fibrillar structures with a number of nodes, which in turn represented a more attractive architecture for focal adhesion.^[^ ^82^ ^]^

Here, it is demonstrated that by applying the numerous concepts within 3D architecture, such as patterning, fibrillar structures, and mesh size, directive cues for specific cellular growth can be created.

Cell spreading, migration, proliferation, and differentiation are essential processes in extracellular matrix synthesis and tissue regeneration. Hydrogel biophysical properties can be used to influence cell behavior and must be finely tuned to achieve the desired cell proliferation, migration, and differentiation. Once the necessary cells are organized in place, the key stage of mineralized matrix deposition and remodeling can occur.

### 3.3 Bone Tissue Deposition and Remodeling

In normal physiology, bone resorption and formation are in a homeostatic equilibrium, meaning that the rate of bone resorption is matched with the replacement of neotissue in response to mechanical load and strain.^[^ ^83^ ^]^ In vitro, before the point of remodeling, osteoprogenitors are required to be delivered within a hydrogel and allowed to reorganize, resulting in a sequential cascade of biological processes including spreading, migration, proliferation, and differentiation.

All of these processes are highly influenced by a plethora of chemical and mechanical properties, along with degradation and 3D architecture as discussed before in Sections 4.1 and 4.2. Eventually, bone formation occurs through collagen secretion and ECM mineralization, followed by remodeling by osteoclasts.^[^ ^84^ ^]^

Upon tissue maturation, cells begin to interact with the hydrogel and native environment, leading to dynamic reorganization of both hydrogels and cells, which enables both cell proliferation and differentiation, as well as cytoskeletal tension. On top of this, construct morphogenesis begins as cells synthesize and deposit nascent ECM proteins and proteoglycans within the hydrogel.^[^ ^85^ ^]^ Within the first 24 h, these nascent proteins can mask hydrogel properties and influence cell–hydrogel interactions within the extracellular space. The newly secreted proteins within the ECM can cause much of the initial, and proceeding, cell–hydrogel interactions to be lost,^[^ ^85^ ^]^ highlighted by studies that show MSCs’ ability to escape and override matrix stiffness cues to remain quiescent.^[^ ^86^ ^]^

Newly secreted proteins and proteoglycans can influence cell–hydrogel interactions in two ways,^[^ ^87^ ^]^ first, by acting as a physical barrier by separating the cell from the hydrogel, and second, by permeating within the engineered hydrogel.^[^ ^87^ ^]^ Understanding and being able to control the latter will influence the outlook of hydrogel design.

The role of the deposited nascent protein is commonly overlooked when studying cell–hydrogel interactions, despite their newly acquired biochemical and biophysical influences which can be quite dissimilar to those originally engineered.^[^ ^85^ ^]^ Little work has been carried out to study how the newly secreted matrix affects the cell–hydrogel interface on local, intermediate, and long‐term interactions. It is suggested that the lack of research in this area can be attributed to limitations in visualizing and measuring secreted matrix dynamics.^[^ ^87^, ^88^ ^]^ The spatiotemporal aspects of ECM assembly and remodeling remain unanswered, and better comprehension is necessary to contribute to our understanding of how cell–hydrogel interactions regulate matrix synthesis and tissue morphogenesis.^[^ ^89^ ^]^ Hydrogel design in the future should pay increased attention to the timing (non‐linear elastic nature of tissue) and accessibility of engineered hydrogel biochemical and biophysical signals on cells.^[^ ^87^ ^]^

At the point of collagen secretion and ECM mineralization, the hydrogel must degrade at an appropriate rate to facilitate neotissue formation, thus hydrogel degradation properties dominate in this point of BTE and will be discussed first.

#### 3.3.1 Role of Hydrogel Degradation and 3D Architecture

Hydrolysis and enzymatic proteolysis are two of the main mechanisms by which hydrogels undergo degradation. Both natural and synthetic hydrogels require incorporation of MMP substrate crosslinkers to allow and aid cell‐triggered proteolysis. During remodeling, MMPs digest the hydrogel to make way for cells to form a new matrix.^[^ ^90^ ^]^ A PEG‐based hydrogel has been used to exploit this property, which, in the presence of cell‐secreted MMPs, was able to deliver bone morphogenetic protein 2 (BMP‐2).^[^ ^91^ ^]^ The process resulted in the formation of bony tissue and mimicked the matrix remodeling observed in natural ECM. Additionally, proteolysis has been used to control the degradation kinetics of a PEG‐based hydrogel from <12 h to over 9 days.^[^ ^92^ ^]^ This was achieved by using the proteolytic enzyme urokinase plasminogen activator to control the rate of peptide bond hydrolysis. In turn, the degradation was responsible for a loss in hydrogel architecture. Degradation in combination with architecture plays a key role in endothelial network formation (Figure 4c).^[^ ^92^ ^]^ Endothelial network formation was only observed in the PEG‐based hydrogels with rapid proteolytic cleavage kinetics that could fully disrupt the infiltrated hydrogel architecture.

Hydrogel network structure and mesh size are key architectural components that influence neotissue formation. Architectural cues in scaffolds play a particular role in regulating vascularization and hence are important within bone remodeling. A hydrogel with open pores and a fully interconnected microchannel network, as small as 40 µm, was created by removing uncrosslinked gel within the structure by soaking the scaffolds in water.^[^ ^93^ ^]^ To achieve a controlled architecture, piezo inkjet 3D printing was used to create an architecture that facilitated vessel formation in vivo following implantation of endothelial cell‐seeded scaffolds.^[^ ^93^ ^]^ Although this study demonstrated vascularization in wound models, the technique and process can also be applied to bone tissue and demonstrates the importance of 3D architecture on vascularization.

Nanoscale symmetry and disorder patterns, in the form of nanocues, have been shown to act as biologically active designs.^[^ ^94^ ^]^ When highly ordered, disordered, and totally random topographies were compared, a disordered square array resulted in significantly higher expression of OPN and osteocalcin (OCN), along with dense aggregates, reminiscent of bone nodule structures.^[^ ^94^ ^]^ This study outlines the important role of different nanotopographies, as well as how developing various orders of design can elucidate specific cell functions.

It is clear from these studies that when designing a hydrogel, degradation and 3D architecture are key considerations to be taken together, not only because of their impact on cell behavior but also because of their close interplay, which in turn can affect cell function for tissue maturation.^[^ ^95^ ^]^

#### 3.3.2 Role of Hydrogel Mechanics

Bulk matrix stiffness has been shown to exhibit a marked dependence on new bone formation in vivo, with intermediate stiffness gels (60 kPa) demonstrating optimal regeneration.^[^ ^56^ ^]^

Two gelatin methacryloyl (GelMA)‐based hydrogels with differing stiffnesses, both co‐cultured with primary human adipose‐derived stem cells (hASCs) (osteogenic) and primary human dermal microvascular endothelial cells (HDMECs) (angiogenic), demonstrated the interplay between HDMECs and hASCs in co‐culture. This study showed successful direction of cell fate when exposed to hydrogels of differing stiffness.^[^ ^96^ ^]^ After 14 days of culture, the stiffer hydrogels induced the hASCs to express Col I and fibronectin, as well as OPN and ALP – key matrix proteins and bone‐specific proteins. Additionally, co‐culture resulted in higher viscoelastic properties of the bone gels than monoculture.^[^ ^96^ ^]^ Conversely, the softer gels induced vascularization while co‐culture promoted a significant increase in stability and complexity of vascular networks. As stated in Section 3, successful vasculature within these hydrogel constructs remains a hurdle in achieving tissue mimics for BTE. However, this work demonstrates that stiffness and co‐culture can be combined and exploited so that the cells can function well in the necessary parts for bone formation and vascularization.

This section has demonstrated how the properties of biomaterials can be finely tuned to direct cell behavior locally to individual cells. Additive manufacturing, specifically biofabrication, is well‐matched to extend biophysical cues from a local to an organ level. Biofabrication could overcome the macro‐level spatial and mechanical complexity by introducing a top‐down approach to create a complex, varying architecture that is analogous to bone and could provide a solution for the diverse spatial arrangement to give tissue‐matching mechanical properties.^[^ ^97^ ^]^ Therefore, the following section focuses on the use of 3D bioprinting for creating architectural intricacy.

## 4 Bioprinting for Bone Tissue Engineering

Bioprinting is one of several approaches for producing constructs that closely mirror the anisotropic complex nature of tissues and organs.^[^ ^98^, ^99^, ^100^ ^]^ The basic principles of bioprinting involve the precise positioning of biological molecules, biomaterials, and cells in a complex 3D architecture with spatial control of the constituents.^[^ ^101^ ^]^ Within bioprinting, there are different manufacturing strategies, including droplet‐based, extrusion‐based, laser‐assisted printing, and stereolithography. The different bioprinting techniques and their advantages and limitations are summarized in **Table** **1**. For detailed reviews of these techniques, the reader is referred to.^[^ ^102^, ^103^, ^104^, ^105^ ^]^ Bioprinting techniques can be used alone or in combination with each other to reach the goal of tissue fabrication.

**Table 1:** Overview of popular 3D bioprinting techniques, and their advantages and limitations

Method	Techniques	Resolution	Speed	Advantages	Disadvantages	References
Droplet‐based	Inkjet bioprinting, including thermal, piezoelectric, and electrostatic	50 µm	Fast (>1 Mbit/s)	Multi‐material, high‐throughput (1–10 000 droplets s^−1^), low viscosity bioinks, and relatively high cell viability	Low upper limit for viscosity of bioink (3.5 and 30 mPaˑs), no overhang, and low cell density	[9, 106, 107, 108, 109, 110]
Pressure‐based	Extrusion bioprinting, including pneumatic, piston‐driven, screw‐driven, co‐axial, and support bath	200 µm	Slow (<1 kbit s^−1^)	Wide range of printable materials, higher viscosity materials (30‐6 × 10^7^ mPaˑs), higher cell densities, multimaterial with co‐axial, and scalability	Low resolution, limited accuracy of patterning, slow printing (10–50 µm s^−1^), shear stress‐related forces on cells, and strut breakup and clogging.	[23, 110, 111, 112, 113, 114, 115, 116]
Laser‐assisted	Laser‐induced forward transfer (LIFT)	10–50 µm	Medium (1 kbit s^−1^ –1 Mbit s^−1^)	Orifice‐free, non‐contact, avoids clogging and shear stress, and high cell viability (>95%)	Expensive, limited scalability, complex to use, and potential thermal injury of hydrogel and cells	[102, 106, 110, 117, 118, 119, 120, 121]
Light‐based	Photolithography, including stereolithography (SLA), digital light processing (DLP), two‐photon polymerization (TPP), and volumetric bioprinting	SLA: 5–300 µm DLP: 50 µm TPP: <100 nm Volumetric: 300 µm	Fast (up to >1 Mbit s^−1^)	Large range of material viscosities, fine geometric features, orifice‐free, non‐contact, and fast printing	UV light risks cell damage, issues with hollow structures, photoinitiators can have cytotoxic effects	[9, 110, 116, 117, 122, 123, 124, 125, 126, 127, 128, 129, 130, 131, 132, 133, 134, 135]

This section focuses on how 3D bioprinting techniques have been used recently for innovation within tissue fabrication. We introduce current challenges in tissue fabrication and recent advances that are overcoming these challenges. We also introduce key innovations in bioprinting which are set to drive future developments in tissue engineering.

### 4.1 Challenges in Fabricating Tissue

There are many challenges associated with in vitro fabrication of tissues and organs, including the supply of cells, requirement for novel biomaterials, and fabricating conditions that match the structural and functional properties of complex and hierarchical tissues and generating vasculature. Bioprinting has the potential to address these challenges by advancing the development of large organs (tens of cm scale) with matching structural and compositional complexity and anisotropy, and blood vasculature while maintaining high cell viability. Thus, challenges to overcome are:

• Fabricating large constructs – print speed/feed rate
• Matching complex and anisotropic structure of tissues – print resolution and multi‐material printing
• Fabricating blood vasculature – creating lumen and hollow fibers
• Maintaining high cell viability – mechanical and photo‐induced damage

An in‐depth understanding of the physical processes involved in the different 3D bioprinting technologies is required to control and manipulate the print speed, resolution, shape, size, and forces involved. There is a complex interplay between these parameters and bioinks to result in a printed construct and so they should be considered in combination.

In addition, the type, source, and density of cell population employed in 3D bioprinted bone constructs for in vitro application is also crucial to consider. Undifferentiated stem cells (e.g., mesenchymal stem cells (MSCs)) have been used extensively due to their unique qualities, such as the capacity for self‐renewal and ability to differentiate into multiple cell lineages. Commonly used stem cells include hASCs,^[^ ^136^, ^137^ ^]^ hMSCs,^[^ ^138^, ^139^ ^]^ and bone MSCs (bMSCs).^[^ ^140^, ^141^ ^]^ Recently, induced pluripotent stem cells (iPSCs) have been employed within bioprinting. These can be derived from most adult cells whilst also allowing differentiation toward MSCs.^[^ ^142^ ^]^

### 4.2 Bioprinting Anatomic Centimeter‐scale Constructs

**Figure 5:**
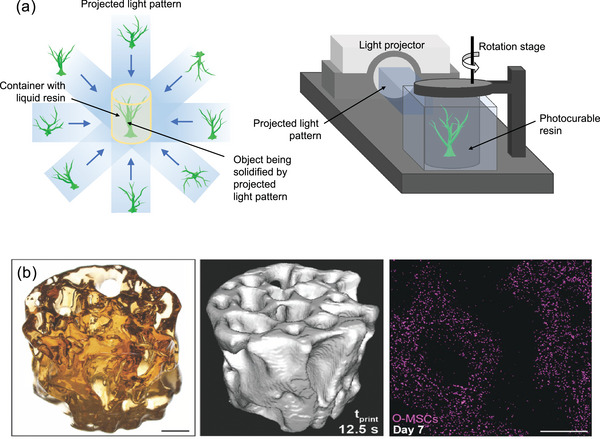
a) Schematic of tomographic projection volumetric printing. b) A printed trabecular bone construct (scale bar = 2 mm) (left), 3D rendering of µCT data (middle), and confocal imaging of the MSC (pink)‐laden construct after culturing for 7 d (scale bar = 1 mm) (right). Reproduced with permission.^[^ ^125^ ^]^ Copyright 2019, Wiley‐VCH GmbH.

Volumetric bioprinting, a relatively new layer‐less additive manufacturing technique, presents a promising solution to constructing centimeter‐scale complex structures at a very fast speed (tens of seconds) without the need for support materials.^[^ ^123^, ^143^ ^]^ It works by controlling the 3D superposition of light exposure on to photocurable resins through orthogonal^[^ ^143^ ^]^ or tomographic techniques (**Figure** **5**a).^[^ ^123^, ^125^, ^144^ ^]^ Gelatin conjugated with acrylate‐based photocurable molecules has been predominantly used to generate constructs with high cell viability (>85%).^[^ ^125^ ^]^

Levato and colleagues have employed volumetric bioprinting to produce cm‐scale MSC‐laden hydrogel constructs with convoluted and interconnected porous networks resembling trabecular bone (Figure 5b).^[^ ^125^ ^]^ In addition to MSC‐laden constructs, they have demonstrated the volumetric printing of organoid‐laden constructs which subsequently matured into liver‐like tissues.^[^ ^145^ ^]^ The printed construct resolution is dictated by both optical and chemical phenomena.^[^ ^126^, ^146^ ^]^ Currently, 85 µm structures can be printed with conventional resins whilst cytocompatible resins allow ≈150 µm resolution.^[^ ^125^, ^126^ ^]^ New resin chemistries^[^ ^144^ ^]^ and setups, such as dual color volumetric printing,^[^ ^147^ ^]^ are being exploited to improve resolution and construct functionality, including stiffness gradients. A major drawback with volumetric printing thus far has been the inability to produce constructs with multiple materials and cell types. Therefore, the printed constructs are currently unable to reproduce the complex anisotropy found in vivo.

### 4.3 Matching Hierarchy and Complex Anisotropy

#### 4.3.1 Multi‐Material and Dual Printing

The emergence of multi‐material printing^[^ ^148^, ^149^, ^150^ ^]^ as well as combining several 3D bioprinting techniques^[^ ^20^, ^151^ ^]^ has advanced the complexity of printed constructs by achieving high‐resolution centimeter‐scale prints with biomimetic and biochemical functionality. Here, we present examples of such advances where researchers have used various 3D printing techniques including extrusion, 2‐photon polymerization, melt‐electro writing, and ink‐jet printing to produce complex structures.

**Figure 6:**
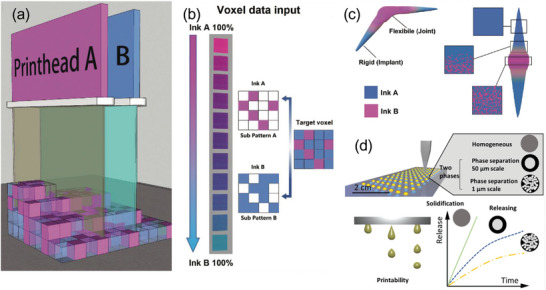
a) Dual inkjet printing unit using UV light to initiate polymerization post‐deposition, b) A randomized printing strategy with complementary patterns – where each was the inverse of the other, c) A printed finger joint implant. Reproduced (Adapted) under the terms of the CC‐BY license.^[^ ^152^ ^]^ Copyright 2021, the Authors. Published by Wiley‐VCH GmbH. d) Controlling the microstructure of materials by means of phase separation, using inkjet printing. Reproduced (Adapted) with permission.^[^ ^154^ ^]^ Copyright 2021, American Chemical Society.

Inkjet printing is currently the “go‐to” technology for multi‐material printing. This is because the technique allows for the spatial variation of material composition, and therefore, distinct areas with specific functions, whilst also permitting scale‐up constructs with high resolution.^[^ ^152^ ^]^ Wildman et al.,^[^ ^152^ ^]^ printed a structure with both rigid and flexible performances, with primarily rigid ink deposition, followed by a flexible ink “filling in” the gaps within the structure (**Figure** **6**a–c). The composite material possessed a range of moduli (1.2 MPa to 2300 MPa) and thus, achieved targeted mechanical responses, with the lowest cell proliferation and less uniform distribution on the flexible ink.^[^ ^152^ ^]^ Inkjet printing has also been used to achieve inks with differing drug release profiles (Figure 6d).^[^ ^153^, ^154^ ^]^ Another study by Wildman et al.,^[^ ^154^ ^]^ used inkjet to successfully control the process of phase separation, which in turn created a microstructure that was exploited to alter the spatial location of drugs and achieve a library of desired drug release profiles.^[^ ^154^ ^]^

Material extrusion additive manufacturing (MEAM) (also referred to as fused deposition modeling (FDM) or fused filament fabrication (FFF)) and extrusion bioprinting approaches have been employed together to deliver and pattern multiple cell‐laden composite hydrogels and/or sacrificial hydrogels. The printing techniques and hydrogels were used together with supporting poly(ɛ‐caprolactone) (PCL) polymer to generate large anatomically relevant tissues.^[^ ^19^ ^]^ Atala^[^ ^19^ ^]^ and Malda^[^ ^149^, ^155^ ^]^ have developed integrated 3D bioprinting workflows to fabricate large, anatomical‐sized multi‐material tissue constructs. The groups employed PCL to achieve mechanical stability and support a host of cell‐laden hydrogels printed to give a complexity approach that was found in vivo.

Development of osteochondral models employing bioprinting approaches with functional hydrogels^[^ ^156^, ^157^, ^158^, ^159^, ^160^ ^]^ to study osteoarthritis has been achieved. The hydrogels, paired with extrusion bioprinting, enhanced the potential of the construct as cell behavior and fate during osteoarthritis onset could be mimicked accurately, demonstrating the power of BTE approach employing hydrogels and bioprinting. Anti‐inflammatory drug targets such as Celecoxib and Rhein have been screened on the inflamed constructs, finding that the drugs were able to downregulate previously upregulated key inflammatory mediators.^[^ ^190^ ^]^

MEAM has also been combined with stereolithography (SLA) to fabricate mechanically strong bone tissue constructs.^[^ ^20^ ^]^ A highly osteogenic bone construct with an organized vascular network was formed through co‐culture of HUVECs and hMSCs. This use of multi‐material and dual printing provides a promising approach for achieving large biomimetic constructs with multiphasic materials. However, the constructs currently suffer from poor resolution and hence are unable to deliver hierarchical arrangements of the different phases below hundreds of microns.

#### 4.3.2 Resolution and Geometry

Two‐photon polymerization (TPP) bioprinting offers one of the best print resolutions of all techniques. This method triggers polymerization solely at the appropriate 3D position using femtosecond laser pulses and two‐photon excitation.^[^ ^161^ ^]^ By moving the focused beam in a photoresist along a computer‐designed 3D route, 3D structures can be created.^[^ ^162^ ^]^ Its capacity to deliver precise architectures has enabled a systematic assessment of construct physical (mesh size) properties on bone‐cell behavior.^[^ ^140^ ^]^ Recently, the capabilities of TPP have been extended to create hydrogel architectures within synthetic cells. Abele et al.,^[^ ^161^ ^]^ demonstrated the ability of TPP to manufacture with high precision and diverse shapes inside preformed giant unilamellar lipid vesicles, including the generation of transmembrane pores to allow the transport of biological cargo. Although TPP gives unprecedented resolution, constructs are limited to millimeters in size.

**Figure 7:**
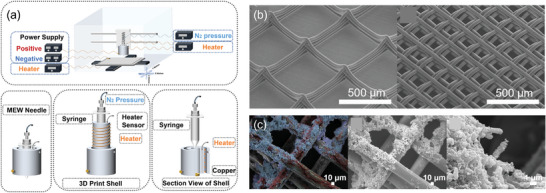
a) Illustrations of the MEW device, including components for high voltage, feeding, heating, direct writing, 3D translation, and sectional views of the spinning head. b) PLLA MEW scaffolds with varying spacing, specifically 500 µm (left) and 200 µm (right). c) Bone formation on the MEW PLLA scaffold after 15 days, represented by false‐color Energy Dispersive X‐ray Spectroscopy (EDS‐SEM) images (with red denoting carbon (C), blue for phosphorus (P), and green for calcium (Ca)) and SEM images of bone growth on the MEW PLLA scaffold. Reproduced (Adapted) with permission.^[^ ^169^ ^]^ Copyright 2021, Elsevier.

Melt electrowriting (MEW) gives architectures with resolutions in the micrometer scale^[^ ^163^, ^164^, ^165^ ^]^ and can be combined with extrusion bioprinting to produce bone tissue constructs. In MEW, molten polymer fibers are drawn onto a computer‐controlled collection plate using electrical fields. Next, repeated fiber‐by‐fiber stacking is used to create 3D constructs (**Figure** **7**a).^[^ ^166^ ^]^ MEW printing is predominantly limited to polyester‐based materials and thus is often used to form high‐resolution mesh‐support structures in bioprinting. However, recent developments such as the printing of biomaterials dissolved in aqueous solutions^[^ ^140^, ^167^ ^]^ and elastomers^[^ ^161^, ^168^ ^]^ are increasing the number of materials amenable to MEW.^[^ ^167^, ^168^ ^]^ Additionally, MEW has been used to fabricate a 3D high‐precision micro‐fibrous polylactic acid (PLA) scaffold with a crosshatch structure and filament diameter of 40 µm and pore size of 200 µm or 500 µm (Figure 7a,b).^[^ ^169^ ^]^ The 200 µm pore‐sized construct demonstrated its in vitro potential for bone formation and integration into the PLA porous structure by 15 days (Figure 7c).^[^ ^169^ ^]^

**Figure 8:**
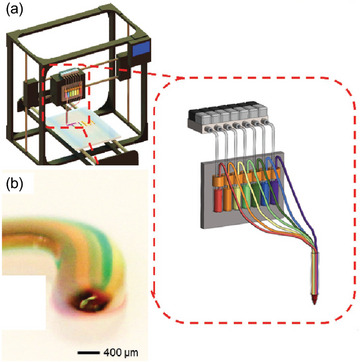
Multi‐material extrusion via digitally tunable continuous extrusion bioprinter. a) Schematic of the multi‐channel printhead together with reservoirs individually actuated by programmable pneumatic valves to deposit b) microfiber containing multiple bioinks. Reproduced with permission.^[^ ^171^ ^]^ Copyright 2017, Wiley‐VCH.

Extrusion bioprinting has been innovatively utilized to produce high material‐resolution structures. Often novel nozzle designs^[^ ^170^, ^171^ ^]^ and fluid mixing^[^ ^172^, ^173^, ^174^ ^]^ have been used to fabricate extruded fibers having high material resolution and complex multi‐material formulations (**Figure** **8**). Yet, these structures require the use of a support material such as PCL fibers or sacrificial layers to allow complex architectures such as overhangs to be printed with soft materials. High‐resolution extrusion printing is possible with stiff and shear‐thinning hydrogels such as Pluronic. However, these do not present an appropriate matrix for cellular processes. Hence, researchers have employed sacrificial printing with stiff glassy materials and Pluronic to fabricate negative woodpile structures that can be removed following casting of the biopolymer hydrogel containing the cells.^[^ ^175^, ^176^ ^]^ The hollow channels left behind provide access to nutrient supply and often enable blood vasculature to form and sprout into the hydrogels once endothelial cells are flowed through. Although this approach allows relatively facile production of cm‐scale 3D constructs using soft cell‐friendly hydrogels, it lacks control over precise positioning of multiple materials and cells. The resulting blood vasculature is also far from the anisotropic structures that are observed in nature.

Finally, stereolithography is often associated with an inability to capture the spatial heterogeneity that controls cell behavior. Project micro‐stereolithography has been used in combination with fluorescent tracers to analyze architectural fidelity and yield precise regional feature alignment.^[^ ^177^ ^]^

#### 4.3.3 Suspended Hydrogels and Vasculature

**Figure 9:**
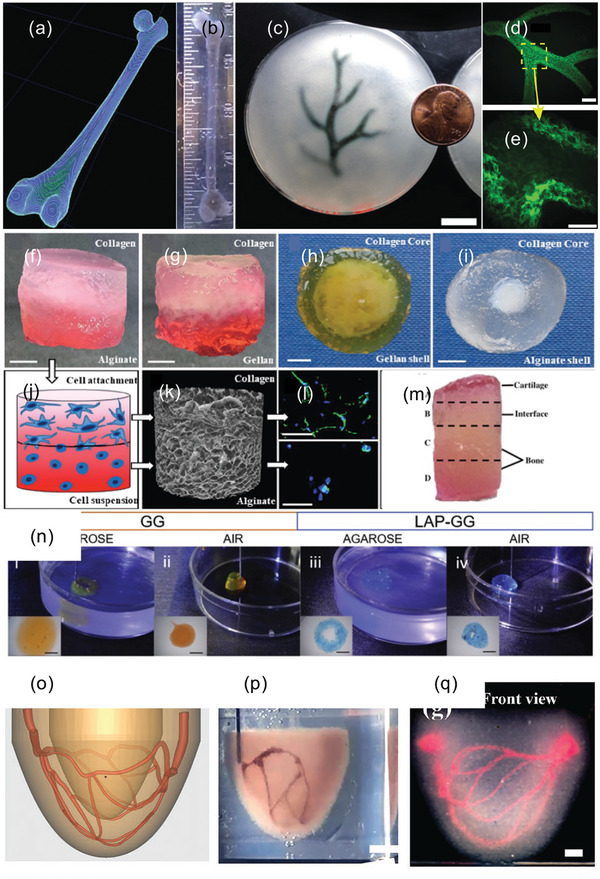
a) A model of a human femur from 3D CT imaging data processed for FRESH printing and b) printed in alginate. c) Human right coronary arterial tree from 3D MRI is processed and FRESH‐printed tree in alginate (black) and embedded in the gelatin slurry support bath. d) Fluorescent alginate (green) of the arterial trees printed in (e) imaged in 3D to show the hollow lumen and multiple branching. Scale bars, (c) 10 mm, (d) 2.5 mm, (e) 1 mm. Reproduced (Adapted) under the terms of the CC0 license.^[^ ^179^ ^]^ Copyright 2015, the Authors. Published by American Association for the Advancement of Science (AAAS). f–m) 3D bioprinting multilayer gradient scaffolds; f,g) bilayer scaffolds and h,i) core–shell scaffolds with j) schematic of cell behavior and attachment, k) micro‐CT, l) Hoechst/actin cell staining of HDFs. Reproduced (Adapted) under the terms of the CC‐BY license.^[^ ^181^ ^]^ Copyright 2019, the Authors. Published by Wiley‐VCH GmbH. n) SLAM – fabrication using either gellan gum (GG) or laponite nanoclay‐gellan gum (LAP‐GG), printed in agarose or air. Reproduced (Adapted) under the terms of the CC‐BY license.^[^ ^18^ ^]^ Copyright 2019, the Authors. Published by Elsevier. o–q) SPIRIT workflow for fabrication of perfusable ventricle construct showing o) 3D design of vascular network, p) image of gelatin ink employed to print the vascular network by extruding into the printed ventricle that served as suspension medium and q) optical image of the 3D‐printed ventricle with a vascular network (perfused with red dye). Scale bars: (p) 5 mm, (q) 2 mm. Reproduced with permission.^[^ ^182^ ^]^ Copyright 2023, Wiley‐VCH GmbH.

Freeform reversible embedding of suspended hydrogels (FRESH),^[^ ^17^, ^178^, ^179^ ^]^ suspended layer additive manufacturing (SLAM),^[^ ^180^, ^181^ ^]^ and sequential printing in a reversible ink template (SPIRIT)^[^ ^182^ ^]^ are forms of support bath bioprinting, employed to achieve stable high‐resolution structures of soft hydrogels and overcome the limitations discussed in Section 5.3.2. The bioink is printed within a self‐healing material, which, during printing, behaves as a liquid in the immediate vicinity of the printing region, allowing for its displacement and deposition of the low viscous bioink. Once the shear is absent, the bath material rapidly reforms to support the extruded bioink prior to gelation. Suspended hydrogels enable fabrication of complex architectures such as vessel branching and closely mimic the anisotropic architecture of the blood vessel wall and cell populations (**Figure** **9**a–e).^[^ ^179^ ^]^ SLAM has been used to print multilayer materials with gradients in structure and chemical stimuli (Figure 9f–m).^[^ ^181^ ^]^ This work demonstrated distinct regional variation within the structure, which ultimately meant cell behavior and interaction could be spatially manipulated. Suspended hydrogel extrusion techniques have also allowed the fabrication of bone constructs using nanocomposites composed of Laponite nanoclay and gellan gum printed within an agarose fluid gel support demonstrating functionality within 1 day which was maintained over 3 weeks (Figure 9n).^[^ ^18^ ^]^ When the nanoclay‐gellan gum was extruded into air, the structure was unstable and unable to support its own weight. Recently, suspension gel method (SPIRIT) has been employed to print complex tissues containing vascular networks (Figure 9o–q).^[^ ^182^ ^]^

Challenges remain within suspended bath hydrogel bioprinting, namely cell distribution during extrusion to avoid clustering, improvement in spatial resolution, and the development of bioinks with a focus on printability, resolution, and stability.^[^ ^183^ ^]^

**Figure 10:**
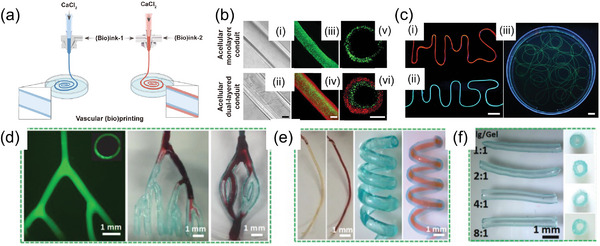
a) Fluidic nozzle extrusion printing of hollow fibers. Schematics of fluidic extrusion of monolayered and dual‐layered vascular conduits. b) Representative lateral‐view bright‐field images i,ii) and fluorescence microscopic images iii,iv), as well as cross‐sectional‐view fluorescence microscopic images v,vi) of monolayered (top) and dual‐layered (bottom) hollow tubes. Scale bars, 200 µm. c) Fluorescence microscopic images of extruded hollow conduits i) an “HMS”‐shaped tube; ii) an “MIT” ‐shaped tube; iii) a randomly placed long tube). Scale bars, 200 µm. Reproduced (Adapted) under the terms of the CC‐BY license.^[^ ^187^ ^]^ Copyright 2022, the Authors. Published by AAAS. d–f) Co‐axial bioprinting of gelatin/alginate scaffolds with fully interconnected microchannels having undergone complete crosslinking, d) fluorescence microscopic image, e) fabricated filaments, f) hollow channel fibers. Reproduced (adapted) with permission.^[^ ^93^ ^]^ Copyright 2022, Elsevier.

Co‐axial bioprinting employing co‐axial and multi‐axial nozzles or microfluidics allows for the simultaneous extrusion of multiple bioinks forming a core and sheath of the extruded hydrogel fibers (**Figure** **10**a–c).^[^ ^184^, ^185^, ^186^, ^187^ ^]^ This approach has been used to produce blood vasculature with a HUVECs core and osteogenic sheath with mouse preosteoblast cells (MC3T3) to generate osteo‐like constructs.^[^ ^188^ ^]^ Advances in microfluidics and controlling the associated hydrogel reactive chemistries during and post‐printing have further advanced the creation of vasculature.^[^ ^93^ ^]^ Luo et al.,^[^ ^93^ ^]^ employed surface crosslinking of a gelatin/alginate hydrogel bioink via divalent cations to create constructs with open macropores and interconnected microchannels whilst providing abundant cell‐recognition sites on gelatin^[^ ^93^ ^]^ (Figure 10d–f). Microfluidic nozzles could be combined with FRESH and SLAM approaches to create complex bioprinted constructs with blood vasculature.

Multi‐material and dual printing within bone tissue constructs has demonstrated great potential. Although it is clear that these approaches demonstrate the formation of either larger‐sized constructs or vasculature; it is important to merge the printing strategies of these separate entities to produce a single construct.

### 4.4 Maintaining High Cell Viability and Phenotype

**Figure 11:**
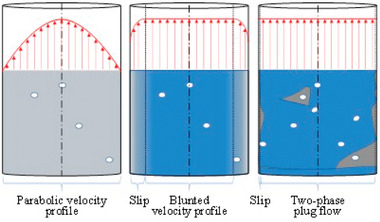
Schematics of the fluid behavior in the Newtonian (left), yield stress or shear‐thinning fluid (middle), and “over‐gelled” (right) bioinks. Reproduced (Adapted) under the terms of the CC‐BY license.^[^ ^191^ ^]^ Copyright 2021, the Authors. Published by Elsevier.

Cell viability depends on the 3D bioprinting technique used. Nozzle‐based methods such as extrusion are direct contact methods and thus, shear stress and extensional forces can lead to reduced cell viability and are considered the main cause of cell damage/death in extrusion‐based 3D bioprinting.^[^ ^113^, ^189^, ^190^, ^191^ ^]^ Shear thinning fluids have the potential to reduce mechanical damage to cells via formation of a plug region shielded by a thin shear‐banding region, which allows fluid flow when shear is applied (**Figure** **11**).^[^ ^191^, ^192^, ^193^, ^194^, ^195^ ^]^ Core–shell printing may also aid in reducing shear on cells.^[^ ^196^ ^]^ However, care must be taken to ensure fluids are truly shear‐thinning and not fracture‐flowing as seen in several “over‐gelled” bioinks.^[^ ^191^ ^]^

Although light‐ and laser‐based methods avoid mechanical damage to cells; damage can still occur through thermal and photo injury, respectively,^[^ ^116^ ^]^ giving rise to potential mutation of the cells. Femtosecond and picosecond lasers with short pulse durations have been used to limit the heat released.^[^ ^120^ ^]^ The effect of laser pulse duration on cell viability has been investigated, by delivering less energy by extending from femtosecond (600 fs) to picosecond (14.1 ps).^[^ ^197^ ^]^ When using optimized conditions, a cell‐survival rate post‐transfer of >95% was achieved across the entire pulse duration range.^[^ ^197^ ^]^ A similar effect is seen with light‐based 3D bioprinting; however, UV light can cause harmful effects (mutagenic, carcinogenic) which can damage cells and affect cell viability.^[^ ^198^, ^199^ ^]^ Therefore, there is a drive toward developing visible light‐based 3D bioprinting techniques instead. Kim et al.,^[^ ^200^ ^]^ developed an SLA system that uses visible light (514 nm) cross‐linkable PEGDA and GelMA hydrogels, and eosin Y as a photoinitiator.^[^ ^200^ ^]^ Upon printing, the 3D constructs achieved a resolution of 50 µm and after 5 days had 85% cell viability.^[^ ^200^ ^]^ Digital light processing (DLP), an extension to SLA, has been applied extensively in BTE^[^ ^104^, ^201^, ^202^ ^]^ with successful demonstration of high cell viability post‐printing with a methacrylated‐silk fibroin bioink using 405 nm light and lithium phenyl‐2,4,6‐trimethyl benzoyl phosphinate (LAP) as photoinitiator.^[^ ^203^ ^]^

Machine learning has been utilized within 3D bioprinting, specifically SLA, to accurately predict cell viability. Using an algorithm, it was concluded that exposure time, followed by layer thickness, GelMA concentration, and light intensity had the greatest effect on cell viability,^[^ ^204^ ^]^, thus allowing accurate prediction of cell viability with respect to different processing parameters. Integrating machine learning into all 3D bioprinting techniques to accurately predict cell viability as well as further outcomes, such as resolution, would be beneficial. The effects of processing parameters on 3D bioprinting on the cellular activity of bioinks are discussed in detail by Adhikari et al.,^[^ ^198^ ^]^

In recent years, there have been numerous advances in 3D bioprinting. These innovations have enabled large tissue and multi‐material printing, with good resolution and improved cell viability. Researchers are continuing to marry these beneficial outcomes, with the ultimate goal to have one printing technology that addresses all of the requirements for in vitro bone tissue scaffolds.

## 5 Concluding Remarks

Formulating novel hydrogels for 3D bioprinting of constructs from BTE with tunable properties has shown significant progress and great potential. However, as tissue engineering is an interdisciplinary field, pivotal challenges remain in the three distinct areas highlighted in this review – BTE, hydrogel design, and 3D bioprinting for advanced manufacturing. To create fully representative in vitro tissue models, which allow long‐term, whole‐construct testing of potential therapeutics, researchers must identify the unmet requirements within these three areas. The first is the dynamic nature and complex architecture of the organ to be recapitulated. The current state‐of‐the‐art in in vitro modeling of bone does not recapitulate the complex hierarchy of bone. This review highlights three main interlinked challenges to BTE, namely complexity, vascularization, and organ‐level simulation. These barriers can be overcome through synergistic developments in hydrogel formulations and merging of bioprinting technologies.

The second is the development and advancement of hydrogel formulations that can recapitulate the stem cell microenvironmental niche and direct the appropriate cell phenotype. This review presents numerous hydrogel biophysical parameters, including stiffness, viscoelasticity, 3D architecture, and nanotopography, which have been successfully modified and controlled, directing stem cell fate. This review highlights the importance of developing novel bioinks employing these hydrogels to bio‐physicochemically cue cells over a time‐scale mapping to key cellular processes in bone formation and remodeling. Advances have been made with dynamically modulating mechanical properties such as stiffness and viscoelasticity, hydrogel network properties, and degradation. However, further advances are required to modulate hydrogel properties in tune with specific cell types and their individual dynamics. Novel hydrogel formulations which combine biopolymers that present natural biochemical signals with synthetic polymers to give reproducible and controlled gelation, mechanics, and degradation, are key.

Finally, 3D bioprinting presents a bridge between hydrogels and achieves the complexity of bone architecture required to facilitate 3D construct development and tissue maturation. Advances in technologies, including volumetric, multi‐material, and microfluidics‐based printing have tremendously improved printing speed, resolution, and complexity. However, these developments are demonstrated with many different bioprinting techniques, and therefore, technological advances to bring these various techniques together without compromising on their merits are key. In addition, progress in areas such as scalability and standardization, without compromising current developments is also important.

## Conflict of Interest

The authors declare no conflict of interest.
